# Contrasting Associations of Urbanization and Lateral Hydrological Gradients With Functional Structure and Rarity of Spontaneous Plants in River Corridors: Evidence From Changde, China

**DOI:** 10.1002/ece3.74622

**Published:** 2026-10-28

**Authors:** Yuxuan Li, Yuandong Hu, Yumeng Zhang

**Affiliations:** ^1^ School of Landscape Architecture, Northeast Forestry University Harbin Heilongjiang China; ^2^ Institute of Science and Engineering of Ecology in Arid and Semi‐Arid Areas, Xi'an University of Architecture and Technology Xi'an Shaanxi China

**Keywords:** functional rarity, functional structure, lateral hydrological gradient, river corridors, spontaneous plants, urbanization gradient

## Abstract

River corridors play an important role in maintaining the diversity and ecological functions of spontaneous plants. Within urban river corridors, spontaneous plant communities are exposed to both urbanization in the surrounding landscape and lateral hydrological variation between water and land. However, the relative contributions of the surrounding urban matrix and the lateral hydrological position to these community properties have rarely been examined within a common framework. Functional rarity was included because it complements conventional diversity metrics by identifying uncommon functional strategies. We surveyed spontaneous plants along six rivers and one lake in the central urban area of Changde, China, and combined analyses of plant functional traits with structural equation modeling to evaluate the associations of urbanization gradient and lateral hydrological gradient with species diversity, functional structure, and functional rarity. The results showed that species diversity (Shannon–Wiener) and functional diversity (FDis) showed unimodal patterns across urbanization levels, with the highest values at intermediate urbanization. Functional trait space was more clearly separated among lateral habitat types than among urbanization levels. Functional rarity increased with ISA but declined with WD. The structural equation models showed contrasting associations of the two gradients with community properties: higher ISA was associated with greater community‐weighted mean plant height and functional rarity but lower species diversity and FDis, whereas greater WD showed the opposite pattern. Functional rarity was positively associated with FDis in both models. This study provides a transferable framework for comparative research across urban river systems. The framework integrates urbanization at the landscape scale, lateral habitat variation within river corridors, and complementary species, functional, structural, and rarity metrics. The framework is helpful in identifying community patterns that assessments based on a single gradient or a single metric may overlook.

## Graphical abstract

This study examined how urbanization and lateral hydrological gradients shape the functional structure and functional rarity of spontaneous plants in urban river corridors in Changde, China. Using field surveys, functional trait analysis, and structural equation modeling, we found that urbanization mainly affected communities through indirect pathways, whereas lateral hydrological gradients were more directly associated with functional differentiation and functional rarity. These findings highlight the need to maintain hydrological habitat continuity and integrate trait‐based community management with conservation of functionally rare species in urban river corridors.

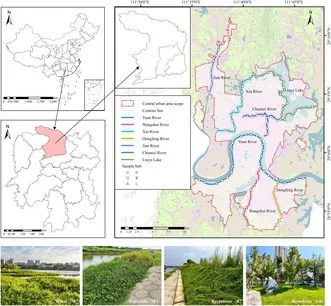

## 1 Introduction

Rapid urbanization is a leading driver of global biodiversity change and has reshaped species composition and ecosystem functioning (Hou, Li, et al. 2023). In urban ecosystems, habitat fragmentation, environmental homogenization, and altered resource distribution frequently result in simplified species assemblages and reduced ecological functioning (McKinney 2008; Aronson et al. 2014). River corridors are characterized by high connectivity and environmental heterogeneity and are considered important for maintaining urban biodiversity (Naiman et al. 2010). However, urban expansion, including channel hardening and altered hydrological processes, has weakened the ecological functions of river corridors and increased the sensitivity of plant communities to disturbance.

Spontaneous plants, defined as plant communities that occur in urban environments without deliberate human intervention, represent an important component of urban vegetation (Bartha et al. 2001; Gao et al. 2025). Owing to their adaptive capacity and responsiveness to environmental variability, spontaneous plants occur across diverse urban spaces, including river corridors (Li, Zhang, et al. 2023), roadsides (Liu et al. 2025), and wastelands (He et al. 2026). These communities contribute to biodiversity conservation, ecosystem services, and community regeneration. Studies of spontaneous plants in river corridors have followed two largely separate lines of inquiry. One line, focused on the surrounding landscape matrix, has examined how urbanization intensity and land‐use patterns are associated with plant diversity and community composition (Li, Li, et al. 2023; Zhang et al. 2023). At the same time, work centered on within‐corridor conditions has emphasized the role of microhabitats and hydrological gradients in shaping community structure (Zhang et al. 2024). While each line has demonstrated that landscape‐level urbanization and local habitat conditions influence spontaneous plant communities, few studies examine both gradients simultaneously. As a result, it remains unclear whether urbanization and lateral hydrological gradients exert similar, contrasting, or interactive effects on the functional structure and functional rarity of spontaneous plant communities.

Trait‐based approaches have increasingly been applied to examine plant responses to environmental gradients and may reflect environmental change more effectively than species diversity alone (Czortek et al. 2020; Eckersley et al. 2024). In urban ecosystems, trait‐based studies of spontaneous plants have shown that urbanization acts as a strong environmental filter, selecting for species with acquisitive nutrient‐use strategies and reducing the representation of functionally distinct species (Hu et al. 2021; Yang et al. 2026). However, these studies have focused on urbanization as a single gradient, leaving the independent role of lateral hydrological processes in filtering community functional composition unresolved. This gap is particularly relevant because urbanization and hydrology may select for different trait dimensions: urbanization tends to favor disturbance‐tolerant, resource‐acquisitive strategies, whereas hydrological gradients select for traits related to waterlogging tolerance and dispersal along moisture gradients.

Recent advances in functional trait ecology have identified functional rarity as an important link between biodiversity and ecological function. Functional rarity integrates functional distinctiveness and local scarcity. By identifying species in unusual positions within community trait space, it complements conventional diversity metrics (Violle et al. 2017). Species with high functional rarity often occupy peripheral niches and have low representation in trait space, but they may contribute to ecosystem multifunctionality and stability under environmental change (Leitão et al. 2016). Empirical work on functional rarity has largely been confined to broad urban land‐use gradients (Sun et al. 2022) or traditional terrestrial systems driven by a single environmental gradient (Walther et al. 2022; Ricotta et al. 2025). River corridors, in contrast, are shaped by two environmental gradients that operate at different spatial scales: regional urbanization and local hydrology. How the functional rarity of spontaneous plant communities varies along the urbanization and lateral hydrological gradients in river corridors remains unknown.

Changde City is located in the core area of the Dongting Lake Ecological and Economic Zone and represents a typical plain river network city with dense waterways and interlaced land and water. Under rapid urbanization, distinct urbanization and lateral hydrological gradients have developed, making the region suitable for examining the effects of different environmental gradients on spontaneous plant communities in river corridors. This study focuses on river corridors in the central urban area of Changde. We quantified species diversity (Shannon–Wiener), functional diversity (FDis), community‐weighted mean traits, and plot‐level functional rarity to address two questions: (1) Do the urbanization gradient (ISA) and the lateral hydrological gradient (WD) show contrasting associations with species diversity, functional diversity, and functional rarity of spontaneous plant communities, and are the effects of these two gradients independent or interactive? (2) Which functional traits mediate the relationships between environmental gradients and community functional structure, and do these mediating traits differ between the two gradients? By addressing these questions, this study provides a framework for understanding how multiple environmental gradients are associated with community assembly in urban river corridors and for informing biodiversity conservation and ecosystem management (Figure 1).

**FIGURE 1:**
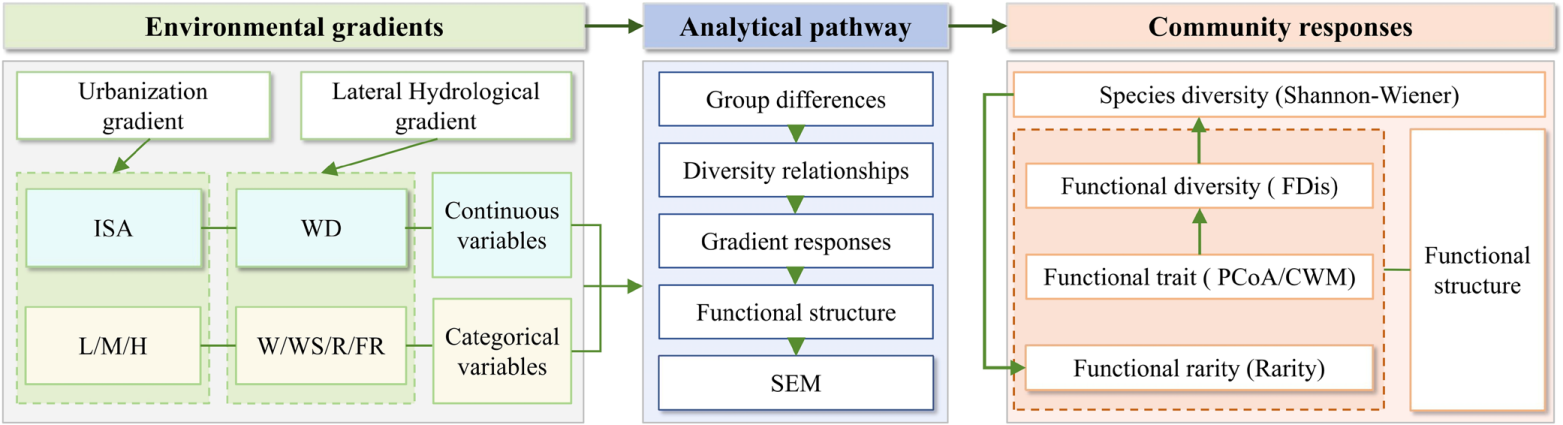
Research framework. The framework integrates two environmental gradients, urbanization and lateral hydrological gradients, with the analytical pathway and community responses. The analytical pathway includes group differences, diversity relationships, gradient response analyses, functional structure analyses, and structural equation modeling. Community responses were evaluated using species diversity (Shannon–Wiener index), functional diversity (FDis), functional traits based on PCoA and community‐weighted means (CWM), and functional rarity (Rarity). ISA and WD represent the two continuous variables. L, M, and H indicate the three urbanization levels (low, medium, high), whereas W, WS, R, and FR indicate the four habitat categories (water, waterside, revetment, and riverfront).

## 2 Materials and Methods

### 2.1 Overview of the Study Area

Changde City is located in northwestern Hunan Province (110°52′–113°51′ E, 27°51′–30°12′N) and has a subtropical humid monsoon climate with distinct seasons and abundant precipitation. The mean annual precipitation is 1352.8 mm (Changde Local Gazetteer Compilation Office 2025), and the mean annual temperature is 17.0°C. The region contains a dense network of rivers and lakes, with the Yuan River and the Lishui River forming the main river systems, together with more than 440 rivers and 1424 reservoirs (Xiong 2019). Groundwater covers 17,600 km^2^, accounting for 96.5% of the total area (Changde Local Gazetteer Compilation Office 2025). Together, these waterways form a well‐connected system of urban river corridors.

Changde is a pilot city for the national Sponge City initiative. Ecological restoration has been carried out in river corridors such as the Chuanzhi River, transforming some heavily polluted drainage channels into urban ecological corridors (Wang et al. 2019; Deng et al. 2022). However, rapid urbanization and the expansion of construction land and impervious surfaces have reduced the naturalness of river corridor shorelines and increased habitat fragmentation (Tao et al. 2024). At the same time, channelization and shoreline hardening have weakened lateral hydrological connectivity between rivers and riparian zones, reduced water‐land ecotones, and weakened the regulation of hydrological processes on spontaneous plant communities (Mason et al. 2025).

The Chuanzi River connects Liuye Lake with the Yuan River, and the Xin River flows into Liuye Lake, together maintaining regional hydrological connectivity and regulation. Based on river connectivity and spatial distribution within the central urban area, six rivers were selected as representative river corridors: the Yuan River, Jian River, Wangshui River, Dongfeng River, Chuanzi River, and Xin River. Liuye Lake was also included because of its close hydrological connections with these rivers. The total length of the river corridors and Liuye Lake is approximately 106.90 km. Together, these waterways form an interconnected river–lake system across areas with different levels of urbanization, forming an urbanization gradient from peripheral to central urban areas (Figure 2).

**FIGURE 2:**
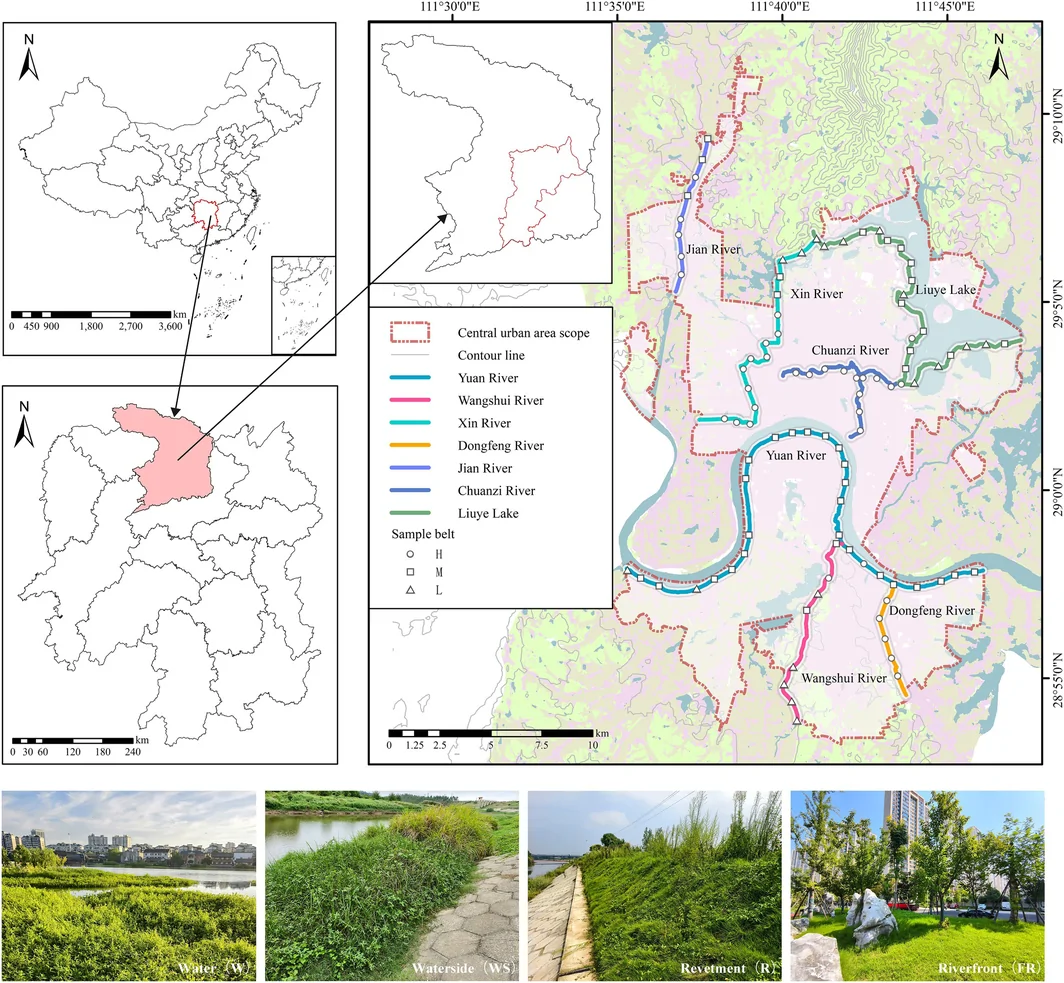
Overview of the study area. The maps show the location of Changde City in China, the sampled river corridors, Liuye Lake, and the sampling belts representing different gradient levels. The red dashed lines indicate the boundary of the central urban area, and the gray lines represent contour lines. Lines in different colors indicate the Yuan River, Wangshui River, Xin River, Dongfeng River, Jian River, Chuanzi River, and Liuye Lake. The circles, squares, and triangles represent sampling sites at high, medium, and low urbanization levels, respectively. The photographs show four representative habitat types: Water, Waterside, Revetment, and Riverfront, abbreviated as W, WS, R, and FR, respectively.

### 2.2 Data Collection

#### 2.2.1 Delineation of the Urbanization Gradient

The urbanization gradient was quantified using high‐resolution Gaofen‐2 satellite images acquired during the summers of 2023 and 2024. Images were ortho‐rectified, radiometrically corrected, and mosaicked before analysis. Construction land patches, including roads, were extracted within river corridors in Changde according to the Current Land Use Classification (GB/T 21010‐2017). A 500 m radius buffer was established around each sampling point, and impervious surface area (ISA) was calculated as the proportion of impervious surfaces within each buffer. The ISA value of each buffer was assigned to all plots within the corresponding transect, and additional buffer‐scale sensitivity analyses were conducted as robustness checks. ISA was used as a continuous measure of urbanization following the urban–rural gradient framework proposed by McKinney and further classified ISA into three levels: low (L, < 20%), medium (M, 20%–50%), and high (H, > 50%) (McKinney 2002).

#### 2.2.2 Vegetation Survey

Field surveys of spontaneous plants were conducted from August to October 2025. Transects were arranged perpendicular to the river at intervals of approximately 1 km. Each transect extended up to 30 m from the waterline. Water distance (WD) was recorded as a continuous variable of the lateral hydrological gradient. Habitats were further classified into four types based on habitat position and characteristics: water (W), waterside (WS), revetment (R), and riverfront (FR). This classification reflects changes in the hydrological gradient from water to land (Figure 2). When the riparian zone was narrow or truncated by infrastructure, transects extended only to the habitat edge, and the actual surveyed length was recorded (Yue et al. 2026). Geographic coordinates, elevation, slope, aspect, and woody cover were also recorded. Plots were established according to vegetation structure within each transect. Given the spatial distribution of the sampling plots, potential spatial dependence was assessed using Moran's I, spatial generalized least squares, and Moran's eigenvector maps (MEMs). Tree communities were surveyed using 10 m × 10 m plots when trees were present. Shrub communities were surveyed using 5 m × 5 m plots, and herb communities were surveyed using five 1 m × 1 m plots located at the four corners and center of each sampling area. In total, 96 transects and 1144 plots were surveyed.

#### 2.2.3 Functional Traits

Given the difficulty of accurately quantifying the number of individuals for herbaceous species, forward selection (ordiR2step) and factor analysis for mixed data (FAMD) were used to select key functional traits and evaluate trait collinearity. The final traits included ecotype, leaf texture, dispersal mode, pollination mode, and plant height (Table 1). These traits reflect ecological strategies related to habitat tolerance, dispersal, reproduction, and competition, thereby helping to explain plant community responses to increasing impervious cover. Trait data were obtained from Flora of China, China Plant Trait Database v2, TRY v6.0, Chinese Colored Weed Illustrated Book, and Seed Information Database (SID). Species data unavailable from online databases were supplemented using published references and field observations (Flora Committee of Chinese Academy of Sciences 2013; Wang et al. 2022; Kattge et al. 2020; Institute for the Control of Agrochemicals, Ministry of Agriculture of the People's Republic of China 2000; Society for Ecological Restoration, International Network for Seed Based Restoration, and Royal Botanic Gardens Kew 2023).

**TABLE 1:** Key functional traits of spontaneous plants and corresponding data sources.

Trait	Type	Value/Range	Source
Ecotype	Nominal	Xerophytic, Mesophytic, Hydrophytic, Aquatic	Liu et al. (2019), Hou, Zeng, et al. (2023)
Leaf texture	Nominal	Membranous, Herbaceous, Papery, Fleshy, Leathery	Flora of China, The China plant trait database version 2
Pollination mode	Nominal	Entomogamous, Anemophilous, Selfed	Plant Trait Database (TRY v6.0)
Dispersal mode	Nominal	Autochory, Zoochory, Anemochory, Hydrochory	Plant Trait Database (TRY v6.0), Chinese Colored Weed Illustrated Book, Seed Information Database (SID)
Plant height (m)	Continuous	0.02–50	Flora of China, field investigation

*Note:* Trait types, values/ranges, and data sources are listed in the table. Additional information for species unavailable from online databases was obtained from published references and field observations.

### 2.3 Data Processing and Statistical Analysis

#### 2.3.1 Calculation of Community and Functional Metrics

Importance value (*P*) was used to represent species dominance in spontaneous plant communities and was calculated as follows:

(1) $$P=\left(H_{i}+C_{i}+F_{i}\right)/3$$

*H*_*i*_ is the relative height, *C*_*i*_ is the relative cover, and *F*_*i*_ is the relative frequency for species *i*. Because spontaneous plant communities in the study area were generally at an early successional stage with weak vertical stratification, the same formula was applied to trees, shrubs, and herbs. The resulting plot‐species importance value matrix was used to calculate diversity indices, with species diversity represented using the Shannon–Wiener index.

(2) $$\text{Shannon}–\text{Wiener}=–\sum P_{i}\ln P_{i}$$

*P*_*i*_ refers to the relative importance value of species *i*.

Because the individual abundance of herbaceous species could not be accurately quantified, relative cover was used as the species weight in the plot‐species matrix (Lavorel et al. 2008; Bishop et al. 2011; Kandlikar et al. 2022). Continuous traits were log‐transformed and standardized before analysis, whereas categorical traits were treated as unordered factors. Functional trait dissimilarity matrices were calculated using Gower distance, and functional diversity within communities was described using functional dispersion (FDis). ISA and WD were standardized using *z*‐scores before subsequent analyses.

(3) $$\text{FDis}=\frac{\sum a_{j}z_{j}}{\sum a_{j}}$$

(4) $$\mathrm{CWM}=\sum_{i=1}^{S}p_{i}\times {\text{trait}}_{i}$$

*a*_*j*_ is the relative cover of species *j* in the community, *z*_*j*_ is the distance from species *j* in the community to the weighted centroid, *p*_*i*_ is the relative cover of species *i* in the community, and trait_*i*_ is the trait value of species *i* in the community.

Functional rarity was calculated from five selected key functional traits. Continuous traits were transformed and standardized before analysis, whereas categorical traits were treated as unordered nominal variables and coded as factors without assigning numerical values or ordinal ranks to trait categories. Gower distances were then calculated from the processed trait matrix to quantify functional dissimilarity among species. Community cover was converted to relative cover before analysis to account for differences in total cover among plots. Functional distinctiveness and species scarcity were normalized to a 0–1 scale and then combined to calculate composite functional rarity.

At the species level, species ranked in the top 10% of overall functional rarity were defined as highly functionally rare species (Grenié et al. 2018; Sun et al. 2022). All remaining species were treated as non‐rare species and used as the comparison group. This classification was used to characterize the composition of functionally rare species and to compare categorical trait composition, including ecotype, leaf texture, pollination mode, and dispersal mode, between highly functionally rare and non‐rare species. Beyond species‐level analysis, this study also weighted the overall functional rarity of species by their relative cover in the plots to get plot‐level functional rarity. This measure was used to analyze changes in functional rarity along urbanization and lateral hydrological gradients, and also served as a response variable in the structural equation model. The relevant calculation formulas are as follows:

(5) $$D_{i}=\frac{\sum_{j=1,j\neq i}^{N}d_{\mathrm{ij}}A_{j}}{\sum_{j=1,j\neq i}^{N}A_{j}}$$

*D*_*i*_ is the functional distinctiveness of species *i*, *D*_*ij*_ is the functional distance between species *i* and species *j*, *A*_*j*_ is the relative cover of species *j* in the community, and *N* is the number of species in the community.

(6) $$S_{i}=\exp\left(–N\times A_{i}\times \ln2\right)$$

*S*_*i*_ is the scarcity of species *i*, *N* is the number of species in the community, and *A*_*i*_ is the relative cover of species *i* in that community.

(7) $$R_{i}=\frac{D_{i}^{'}+S_{i}^{'}}{2}$$

*R*_*i*_ is the overall functional rarity of a species, while *D*_*i*_ and *S*_*i*_ are the standardized functional uniqueness and scarcity, respectively.

(8) $$R_{\text{site}}=\sum_{i=1}^{S}p_{i}R_{i}$$

*R*_site_ represents the functional rarity of a plot scale, *p*_*i*_ is the relative cover of species *i* in that plot, and *R*_*i*_ is the overall functional rarity of species *i*.

#### 2.3.2 Statistical Analyses

Spearman correlation was used to assess the relationship between species diversity (Shannon–Wiener) and functional diversity (FDis). Regression analyses were used to examine variation in FDis and functional rarity along the urbanization gradient (ISA) and the lateral hydrological gradient (WD). Community functional trait space was visualized using principal coordinates analysis (PCoA) based on the Gower distance matrix. Site‐level scores were calculated as centroids weighted by relative cover across all positive PCoA axes. Differences in community functional composition across urbanization levels and habitat types were tested using PERMANOVA on Euclidean distances of site‐level scores. Homogeneity of multivariate dispersion was assessed using betadisper based on the same distance matrix.

Community‐weighted mean traits (CWM) were analyzed to identify key functional traits associated with ISA and WD, focusing on the main CWM variables showing clear responses along these gradients. Differences in categorical trait composition between highly functionally rare and non‐rare species were further compared across urbanization levels and habitat types using Fisher's exact tests with Monte Carlo simulation, and *p* values were adjusted using the Benjamini–Hochberg method.

Structural equation modeling (SEM) was used to evaluate hypothesized pathways among ISA or WD, community‐weighted mean traits (CWM), FDis, Shannon–Wiener diversity, and plot‐level functional rarity. ISA and WD were analyzed in two separate overall SEMs, with woody coverage retained as a vegetation‐structure covariate based on environmental screening and model sensitivity analyses. Continuous variables were standardized, and WD was transformed as ln (WD + 1) prior to standardization. Model fit and multicollinearity diagnostics were assessed for all SEMs. Multi‐group SEMs by urbanization level and habitat type were used as supplementary robustness checks. For each variable, we calculated its relative association magnitude as the absolute standardized total association divided by the sum of the absolute standardized total associations. We quantified uncertainty using 5000 bootstrap samples generated by resampling cases with replacement and calculated percentile‐based 95% confidence intervals.

All statistical analyses were conducted in R. The Shannon–Wiener index and PCoA were calculated using the *vegan* package, FDis using the *FD* package, functional rarity using the *funrar* package, and Gower distance using the *cluster* package. Forward selection was performed using *vegan*, and FAMD was conducted using *FactoMineR*. PERMANOVA was conducted using the adonis2 function in the vegan package. Structural equation models were constructed using *lavaan*, and figures were generated using *ggplot2*.

## 3 Results and Analysis

### 3.1 Patterns of Species Diversity and Functional Diversity Distribution of Spontaneous Plants in River Corridors

A total of 307 spontaneous plant species belonging to 229 genera and 82 families were recorded. Species diversity (Shannon–Wiener) and functional diversity (FDis) differed among urbanization levels (*p* < 0.001). Both indices showed unimodal changes with urbanization and reached their highest values at intermediate urbanization levels (Figure 3A,B). Shannon–Wiener index also differed among habitat types (*p* < 0.001), with the highest values observed in R habitats and the lowest in W habitats (Figure 3C). FDis varied among habitat types (*p* = 0.016), with the highest FDis observed in WS habitats and the lowest in W habitats (Figure 3D). The relatively high FDis in WS habitats suggests that the transition zone between water and land provides a broader range of environmental conditions for species with different functional strategies. Overall, both species diversity and functional structure of spontaneous plant communities differed significantly by the levels of urbanization and habitat types. The urbanization gradient primarily manifested as a nonlinear change in diversity, while the lateral hydrological gradient was reflected in the spatial differentiation of community structure among habitats.

**FIGURE 3:**
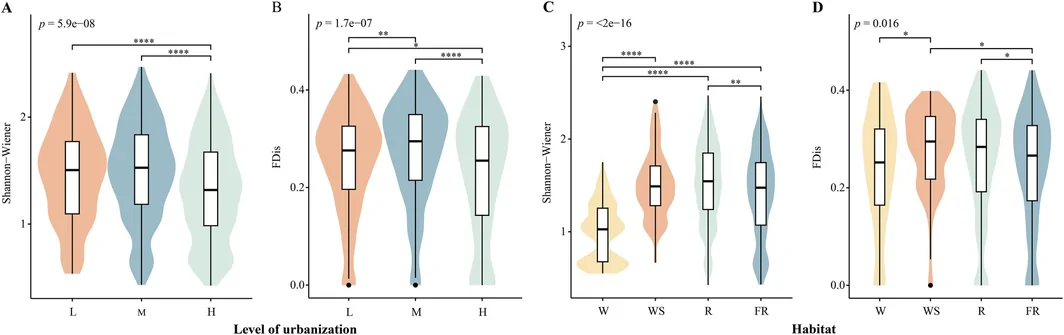
Species diversity and functional diversity of spontaneous plant communities across urbanization levels and habitat types. (A) Species diversity (Shannon–Wiener) across urbanization levels (L, M, H); (B) Functional diversity (FDis) across urbanization levels; (C) Species diversity across habitat types (W, WS, R, FR); (D) Functional diversity across habitat types. Sample sizes (*n*): L = 243, M = 489, H = 412; W = 145, WS = 101, *R* = 568, FR = 330. Asterisks indicate significant differences based on Wilcoxon test results (*p < 0.05, **p < 0.01, ***p < 0.001, ****p < 0.0001).

### 3.2 Relationship Between Species Diversity and Functional Diversity of Spontaneous Plants

The Shannon–Wiener index and FDis were positively correlated across urbanization levels (Figure 4A). The strongest correlation was observed at high urbanization levels (*ρ* = 0.70). This stronger association suggests that species diversity and functional diversity were more closely coupled under higher urbanization pressure. Positive correlations between the Shannon–Wiener index and FDis were also observed across habitat types (Figure 4B). The correlation coefficient was highest in R habitats (*ρ* = 0.65) and lowest in WS habitats (*ρ* = 0.39). Specifically, the consistently positive correlations suggest a general coupling between species diversity and functional diversity, while differences in correlation strength indicate that this relationship was context‐dependent across urbanization and habitat gradients.

**FIGURE 4:**
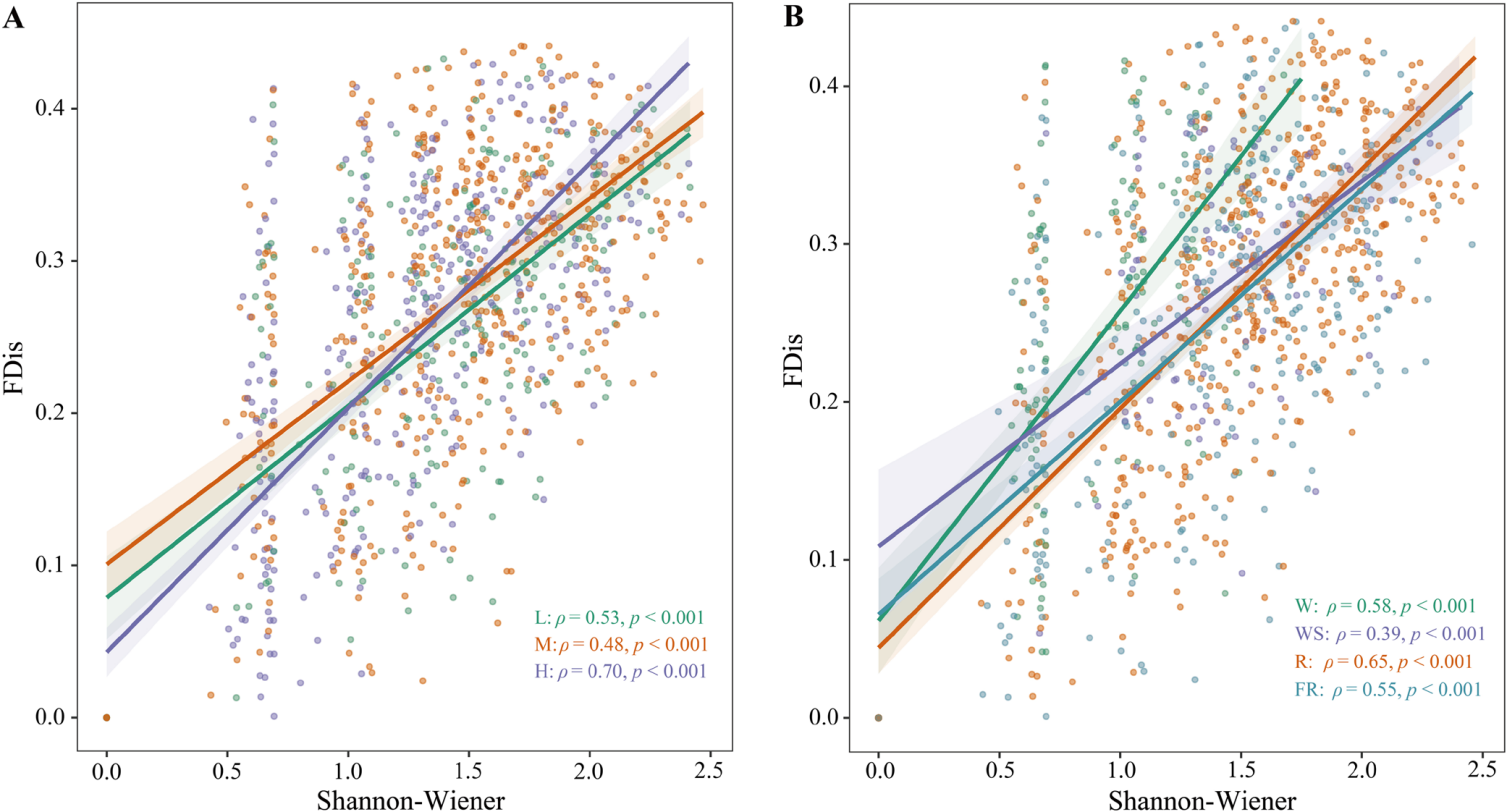
Relationships between species diversity and functional diversity of spontaneous plants across urbanization levels and habitat types. (A) Relationships across urbanization levels (L, M, H; *n* = 243, 489, and 412, respectively); (B) Relationships across habitat types (W, WS, R, FR; *n* = 145, 101, 568, and 330, respectively). Solid lines represent regression fits, and shaded areas indicate 95% confidence intervals.

### 3.3 Functional Structure and Trait Responses Along Urbanization and Lateral Hydrological Gradients

Functional diversity (FDis) showed a nonlinear relationship with the urbanization gradient (ISA), increasing from low to moderate ISA and declining at high ISA levels (Figure 5A). FDis generally increased along the lateral hydrological gradient (WD), suggesting broader functional differentiation from water habitats toward riverfront habitats.

**FIGURE 5:**
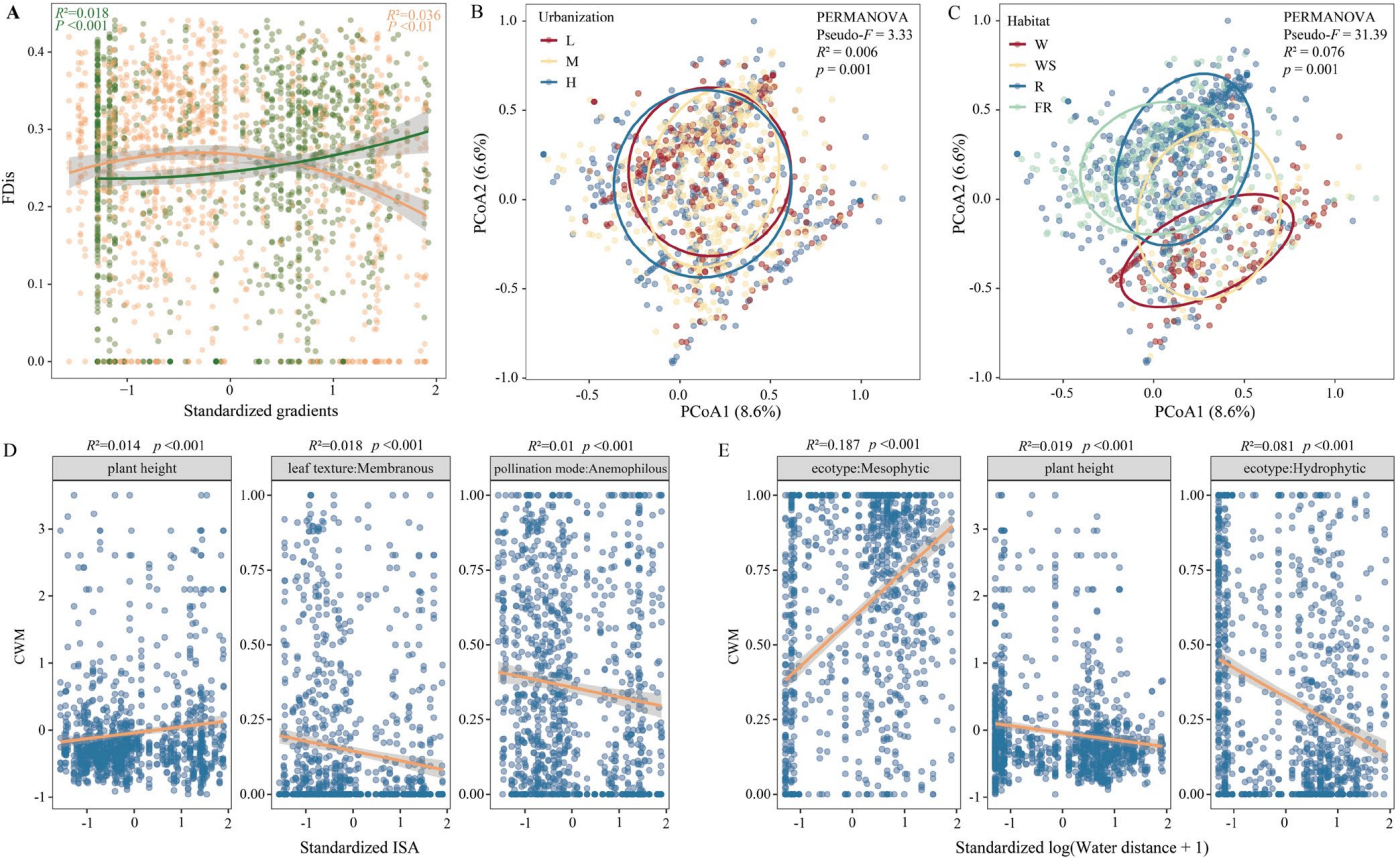
Functional structure and trait responses of spontaneous plant communities along the urbanization gradient and lateral hydrological gradient. (A) Variation in FDis along ISA and WD, with orange representing the urbanization gradient and green representing the lateral hydrological gradient; (B) PCoA of functional trait space across urbanization levels; (C) PCoA of functional trait space across habitat types; (D) Responses of community‐weighted mean traits (CWM) to the urbanization gradient; (E) Responses of community‐weighted mean traits (CWM) to the lateral hydrological gradient. Points represent plots (*n* = 1144), solid lines represent fitted trends, and shaded areas indicate 95% confidence intervals. Group sample sizes were L = 243, M = 489, and H = 412 in (B), and W = 145, WS = 101, *R* = 568, and FR = 330 in (C).

Principal coordinates analysis (PCoA) showed substantial overlap in functional trait space among urbanization levels (Figure 5B). Although PERMANOVA detected a significant difference among urbanization groups (Pseudo‐*F* = 3.33, *R* ^2^ = 0.006, *p* = 0.001), the very small *R* ^2^ indicated that urbanization explained only a limited proportion of the variation in community functional composition. This result should be interpreted cautiously because within‐group dispersion also differed among urbanization levels (*p* = 0.001), suggesting that the PERMANOVA may partly reflect differences in dispersion rather than differences in group centroids alone. By contrast, habitat types showed clearer separation in trait space (Pseudo‐*F* = 31.39, *R* ^2^ = 0.076, *p* = 0.001; Figure 5C), and their within‐group dispersion did not differ significantly (*p* = 0.134).

CWM trait responses further showed contrasting associations with the two gradients. Along the urbanization gradient, CWM plant height increased with ISA, whereas membranous leaf texture and anemophilous pollination mode decreased (Figure 5D). Along the lateral hydrological gradient, CWM plant height decreased with increasing ln (WD + 1), while mesophytic ecotype increased and hydrophytic ecotype decreased (Figure 5E). The contrasting responses of CWM plant height to ISA and WD indicate that plant height captured a key trait‐level distinction between the two gradients, supporting its inclusion as the focal CWM trait in the subsequent SEM.

### 3.4 Functional Rarity and Trait Composition Along Environmental Gradients

Functional rarity of spontaneous plant communities varied across environmental gradients, increasing significantly with ISA (*R* ^2^ = 0.020, *p* < 0.001; Figure 6A) and decreasing significantly with WD (*R* ^*2*^ = 0.017, *p* < 0.001; Figure 6C). Across urbanization levels, the trait composition of non‐rare species was relatively stable and was mainly characterized by mesophytic ecotypes, papery leaves, and entomogamous pollination (Figure 6B). In contrast, highly functionally rare species showed greater compositional variation: their ecotype composition shifted from predominantly hydrophytic types at low urbanization to mesophytic types at higher urbanization, while leaf texture shifted from a relatively high proportion of membranous leaves toward papery leaves. Pollination and dispersal modes showed no consistent directional replacement. Trait composition also varied among habitat types (Figure 6D). The composition of non‐rare species was relatively similar across habitats, whereas highly functionally rare species showed clearer habitat‐associated variation. In W and WS habitats, highly functionally rare species were characterized by relatively higher proportions of mesophytic ecotypes and herbaceous or membranous leaves, whereas in R and FR habitats, they showed relatively higher proportions of hydrophytic ecotypes and papery leaves. These patterns suggest that habitat type was associated with greater variation in the trait composition of highly functionally rare species than urbanization level. It is worth noting that the stacked bar charts provide descriptive summaries of categorical trait composition. After multiple‐testing correction, no overall significant differences in categorical trait proportions were detected among urbanization levels. Across habitat types, only the ecotype composition of non‐rare species differed significantly; the remaining comparisons were not significant after correction.

**FIGURE 6:**
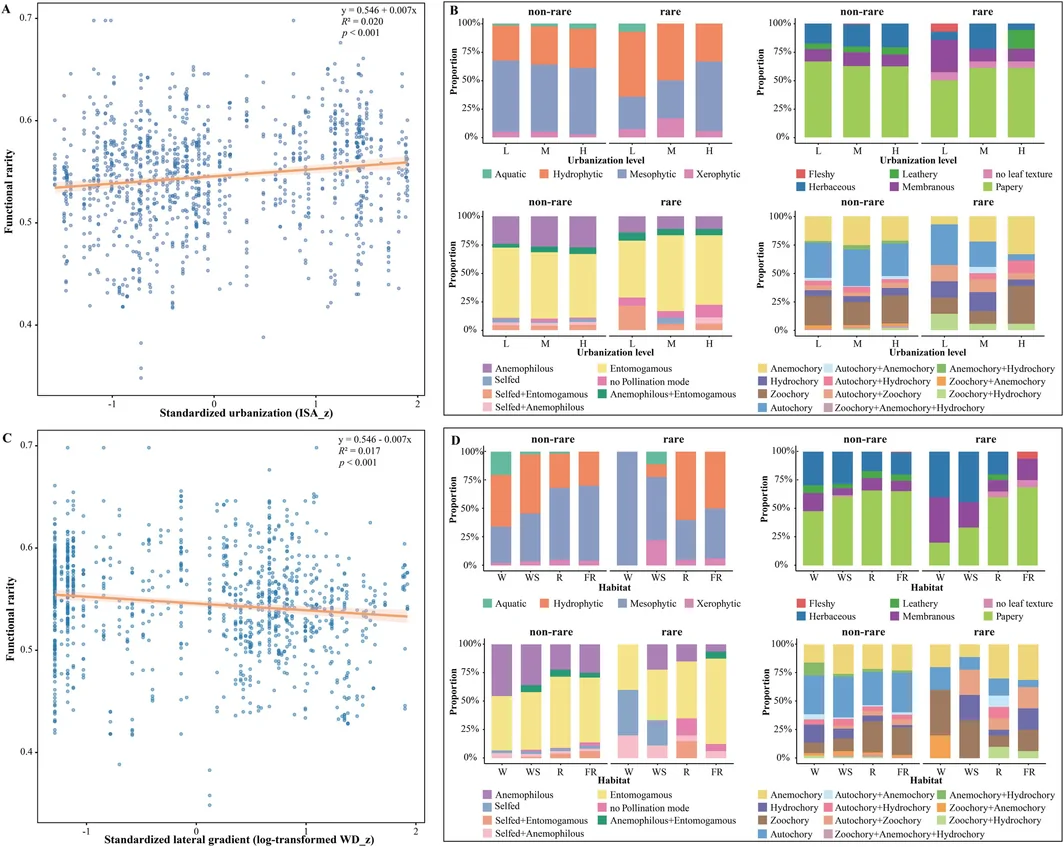
Changes in functional rarity and trait composition of spontaneous plant communities along the urbanization gradient and lateral hydrological gradient. (A) Relationship between functional rarity and the urbanization gradient (ISA); (B) Proportional composition of ecotype, leaf texture, pollination mode, and dispersal mode for non‐rare and highly functionally rare species across urbanization levels; (C) Relationship between functional rarity and the lateral hydrological gradient (WD); (D) Proportional trait composition of non‐rare and highly functionally rare species across habitat types. Points represent plots, solid lines represent fitted regression models, and shaded areas indicate confidence intervals. Sample sizes (non‐rare/rare) were L = 120/14, M = 158/18, and H = 154/18 for urbanization levels, and W = 44/5, WS = 81/9, *R* = 176/20, and FR = 144/16 for habitat types.

### 3.5 Hypothesized Associations Among Functional Structure and Functional Rarity in Spontaneous Plant Communities

The overall SEMs showed contrasting path associations between ISA and WD and community functional responses. The ISA and WD models are presented in Figure 7A,B, respectively. In the ISA model, ISA was positively associated with CWM plant height (*β* = 0.09) and functional rarity (*β* = 0.09), but negatively associated with Shannon–Wiener diversity (*β* = −0.10) and FDis (*β* = −0.09; Figure 7A). In contrast, in the WD model, WD was negatively associated with CWM plant height (*β* = −0.14) and functional rarity (*β* = −0.07), but positively associated with Shannon–Wiener diversity (*β* = 0.22) and FDis (*β* = 0.10; Figure 7B).

**FIGURE 7:**
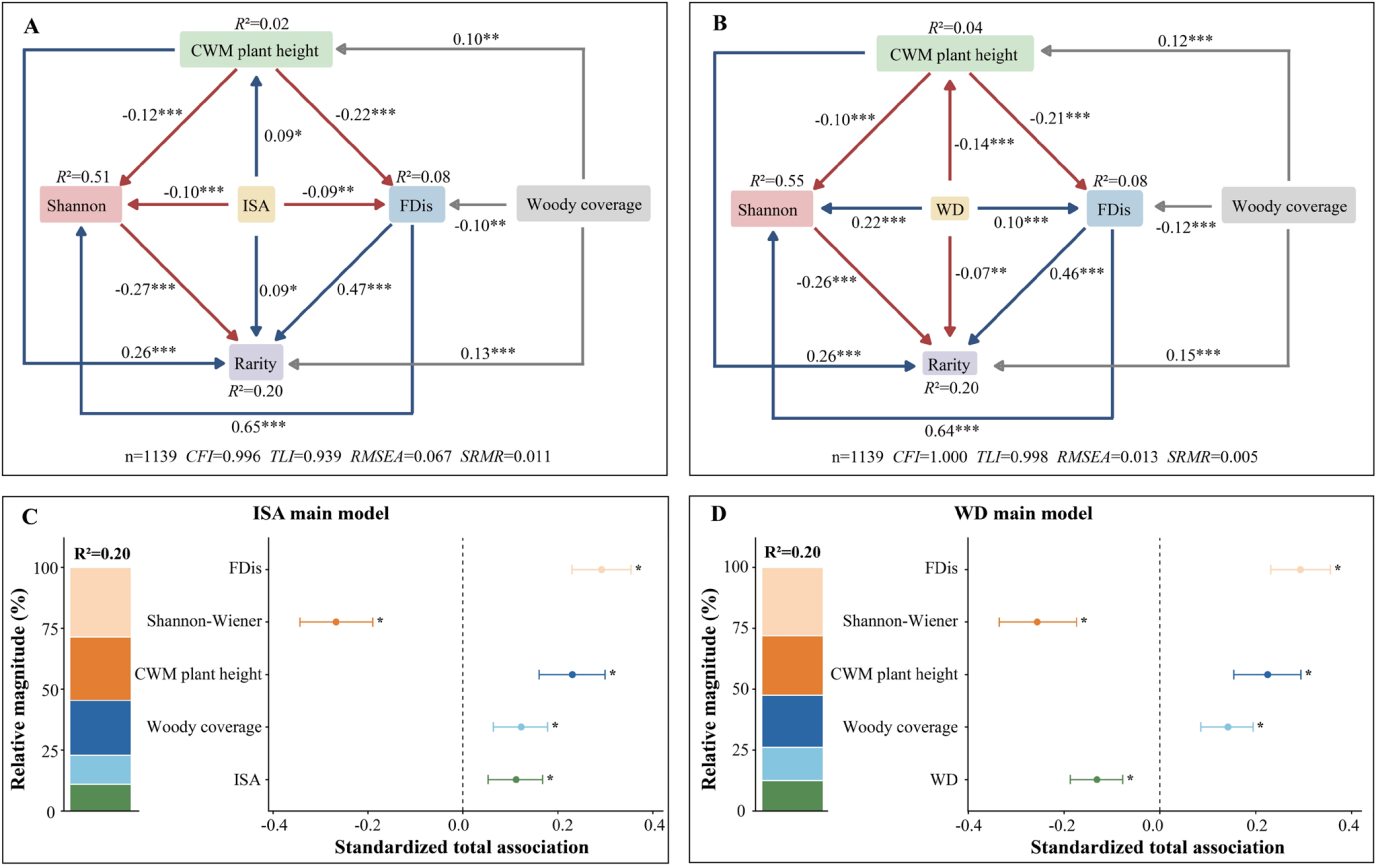
Structural equation models and bootstrap estimates of standardized total associations with functional rarity across urbanization and lateral hydrological gradients. (A) Overall SEM for the urbanization gradient; (B) Overall SEM for the lateral hydrological gradient. (C) Standardized total associations in the ISA main model; (D) standardized total associations in the WD main model. In (A) and (B), arrows indicate specified associations, and values are standardized path coefficients (*β*). Blue and red arrows indicate positive and negative associations, respectively; gray arrows represent associations involving woody coverage as a covariate. *R* ^2^ values indicate explained variance, and *n* indicates sample size. In (C) and (D), stacked bars show the relative magnitude of each variable's absolute standardized total association, while points and horizontal lines show standardized total associations and percentile‐based 95% confidence intervals from 5000 nonparametric bootstrap resamples. Asterisks in (A) and (B) indicate *p < 0.05, **p < 0.01, and ***p < 0.001; in (C) and (D), significant standardized total associations are indicated by an asterisk (*). ISA, impervious surface area; WD, water distance; CWM plant height, community‐weighted mean trait for plant height; FDis, functional dispersion; Shannon, Shannon–Wiener index; Rarity, functional rarity.

In both models, CWM plant height was negatively associated with FDis (ISA: *β* = −0.22; WD: *β* = −0.21), while FDis was positively associated with functional rarity (ISA: *β* = 0.47; WD: *β* = 0.46), whereas Shannon–Wiener diversity was negatively associated with functional rarity (ISA: *β* = −0.27; WD: *β* = −0.26). Woody coverage showed consistent associations in both models, being positively associated with CWM plant height (ISA: *β* = 0.10; WD: *β* = 0.12) and functional rarity (ISA: *β* = 0.13; WD: *β* = 0.15), but negatively associated with FDis (ISA: *β* = −0.10; WD: *β* = −0.12). Thus, higher ISA was associated with greater plant height and functional rarity but lower species and functional diversity, whereas greater WD showed the opposite pattern. The contrasting responses of CWM plant height further indicate that this trait captured differences in community functional responses across the two gradients. The bootstrap‐based standardized total associations also showed that in both models, FDis and Shannon–Wiener diversity represented the largest relative magnitudes of association with functional rarity, whereas ISA and WD showed smaller but contrasting overall associations (Figure 7C,D).

Mixed effects models provided no evidence for ISA × WD interactions across all four response variables (Table 2): Shannon–Wiener diversity (*β* = 0.002, *p* = 0.896), FDis (*β* = 0.003, *p* = 0.432), functional rarity (*β* = 0.001, *p* = 0.568), and CWM plant height (*β* = 0.028, *p* = 0.269). Model comparison further supported the additive structure: incorporating interaction terms consistently increased AIC values relative to the additive‐only models. Taken together, these results indicate that ISA and WD exhibited contrasting but additive functional associations, with no evidence that the association of WD with community functional metrics varied systematically along the urbanization gradient.

**TABLE 2:** Tests of ISA × WD interaction effects on community diversity, functional structure, and functional rarity.

Response variable	*β*_(ISA × WD)_	*t*	*p*	ΔAIC
Shannon–Wiener diversity	−0.002	−0.130	0.896	1.983
Functional dispersion (FDis)	0.003	0.787	0.432	1.381
Functional rarity	0.001	0.571	0.568	1.674
CWM plant height	0.028	1.106	0.269	0.778

*Note:* ISA denotes impervious surface area, and WD denotes water distance. *β*_(ISA × WD)_ represents the estimated coefficient of the ISA × WD interaction term. The *t* statistic and *p* value test whether the interaction coefficient differs from zero. ΔAIC is calculated as the AIC of the interaction model minus the AIC of the additive model. Positive ΔAIC values indicate that adding the interaction term did not improve model fit.

## 4 Discussion

### 4.1 Contrasting Associations of Urbanization and Lateral Hydrological Gradients With Community Functional Structure and Rarity

The urbanization gradient and the lateral hydrological gradient were associated with community functional structure in opposite directions: higher ISA was associated with lower species and functional diversity but higher functional rarity, whereas the lateral hydrological gradient showed the opposite tendency. Community‐weighted mean plant height also responded in opposite directions to the two gradients. These patterns indicate that the two gradients reflect different forms of environmental filtering. ISA represents the surrounding urban landscape matrix, where increasing imperviousness is associated with fragmentation, altered resource conditions, and stronger anthropogenic disturbance (McKinney 2008). Such conditions may favor a narrower set of tolerant strategies and reduce the breadth of functional space occupied by the community, consistent with functional homogenization in urban ecosystems (McKinney 2006). By contrast, WD reflects position within the river corridor, where changing proximity to water is associated with shifts in moisture availability and habitat type (Naiman et al. 2010).

The opposite responses of plant height to ISA and WD further support this interpretation, suggesting different trait‐level selection pressures. The positive association between ISA and CWM plant height is consistent with evidence that urbanization can indirectly promote vegetation growth through altered resource and management contexts (Zhang et al. 2022), and that urban growth can reshape the trait composition of spontaneous plant communities (Hu et al. 2021). In contrast, the negative association between WD and plant height may reflect a shift from near‐water habitats to more managed upland or roadside habitats, where planted vegetation and disturbance can constrain taller spontaneous plants. Similar distance‐related declines in CWM maximum height have been reported in riparian corridors (Maracahipes‐Santos et al. 2020; Kinnoumè et al. 2024). This pattern is consistent with the mass‐ratio hypothesis, which emphasizes the role of dominant trait values in reflecting prevailing environmental conditions (Smith et al. 2020), but direct evidence on management intensity would be needed to confirm these mechanisms. Woody coverage, included as a covariate, provided an additional perspective on local vegetation structure. It was positively associated with CWM plant height and functional rarity but negatively associated with FDis in both SEMs. These contrasting associations highlight the importance of woody coverage as an indicator of local vegetation structure and demonstrate the value of evaluating vegetation structure alongside diversity metrics in restoration assessment (Foley‐Congdon et al. 2024).

The absence of a detectable ISA × WD interaction suggests that the WD‐associated patterns did not vary systematically across the ISA gradient. Previous studies have shown that plant communities in riparian corridors respond differently to the surrounding landscape context and to local corridor conditions (Burton et al. 2009). This supports interpreting ISA and WD as distinct environmental dimensions rather than as interchangeable indicators. Future studies should test whether these contrasting gradient responses hold in other urban river systems and under different climatic and geomorphic settings.

### 4.2 Functional Differentiation and Context Dependence Across the Two Gradients

Habitat types showed clearer separation in functional trait space than urbanization levels, likely because habitat position within the river corridor corresponds to differences in inundation, moisture availability, substrate, and bank conditions. These local conditions correspond to distinct combinations of plant functional strategies along the lateral gradient (Tisserant et al. 2024). In contrast, ISA represents the surrounding urban landscape matrix rather than local conditions within the river corridor. The substantial overlap among urbanization levels therefore suggests that variation in the surrounding impervious‐surface context was associated with relatively modest shifts in community functional composition, without clear separation among different urbanization levels. This pattern is consistent with evidence that local corridor conditions are more closely related to community‐level traits than the surrounding landscape matrix (Schwoertzig et al. 2016). Future studies should combine finer‐scale habitat measurements with broader spatial comparisons to determine whether the relative importance of these different environmental contexts is consistent across river networks.

The relationship between species diversity (Shannon–Wiener) and functional diversity (FDis) varied across environmental contexts. The correlation between Shannon–Wiener diversity and FDis was strongest under high urbanization, suggesting that species turnover in more urbanized sites was more closely accompanied by changes in functional trait space. This pattern reflects lower functional redundancy under stronger environmental constraints, where additional species are more likely to represent distinct functional strategies. Along the lateral hydrological gradient, the relationship was strongest in R habitats and weakest in WS habitats. The stronger coupling in R habitats is also consistent with more constrained conditions, whereas the weaker coupling in WS habitats is consistent with dynamic water‐land transition zones in which flooding, dispersal, and local disturbance weaken the correspondence between species occurrence and functional differentiation. These findings are consistent with previous studies showing that the relationship between species diversity and functional diversity varies along environmental and disturbance gradients (Biswas and Mallik 2011; Cadotte et al. 2011; Wang et al. 2025).

The trait composition of functionally rare and non‐rare species varied descriptively across habitats, whereas statistical support for context dependence was limited. Across habitat types, only the ecotype composition of non‐rare species differed significantly after multiple‐testing correction. This pattern is ecologically plausible because ecotype is directly related to habitat moisture conditions and therefore more likely to vary along the lateral hydrological gradient (Silvertown et al. 2015; Ran et al. 2022). Non‐rare species also had larger sample sizes and more stable occurrence across habitats, making broad habitat‐related shifts easier to detect statistically. In contrast, functionally rare species occurred in smaller numbers and were more unevenly distributed, which may have reduced the power to detect compositional differences even when descriptive variation was visible in the stacked bars. Therefore, the stacked‐bar patterns should be treated as descriptive evidence of potential trait variation rather than strong statistical support for trait replacement.

In both SEMs, functional rarity increased with functional diversity (FDis) but decreased with species diversity (Shannon–Wiener). This consistent pattern indicates that functional rarity captures community properties distinct from species diversity and functional diversity. Previous studies have primarily examined species diversity, functional diversity, or their relationship (Biswas et al. 2019; Flores‐Galicia et al. 2021; Eckersley et al. 2024). By integrating the representation of peripheral trait combinations with the density of species occupancy in functional trait space, functional rarity characterizes the retention of uncommon functional strategies across environmental contexts (Leitão et al. 2016). Communities with broader functional space can retain more peripheral trait combinations, whereas greater species richness can reduce the relative distinctiveness of individual species by filling trait space more densely. Functional rarity therefore complements conventional species and functional diversity metrics and strengthens the assessment of spontaneous plant communities in urban river corridors.

### 4.3 Implications for Multi‐Gradient Assessment and Management of Urban River Corridors

The contrasting responses of plant diversity, functional structure, and functional rarity to urbanization and lateral hydrological gradients indicate that urban river corridors cannot be adequately assessed using a single gradient or a single diversity metric. Instead, ecological assessment should integrate species diversity, functional dispersion, functional composition, functional rarity, and community‐weighted mean plant height. Integrating these complementary metrics captures the multifaceted dynamics of vegetation across species composition, functional traits, and structural properties (Gallardo et al. 2011). In particular, plant height may provide a readily interpretable indicator of vegetation structure, while functional dispersion and functional rarity may help identify changes in functional differentiation. Such multidimensional assessment is consistent with calls for using multiple vegetation indicators in riparian restoration monitoring (Fraaije et al. 2019; Cupertino et al. 2024). The value of plant height as an assessment indicator depends on local environmental and management contexts. Its relevance should therefore be evaluated alongside site‐specific hydrological, habitat, and vegetation conditions rather than assumed to apply uniformly across urban river corridors. Further studies across a wider range of river systems are needed to assess the generality of its association with functional structure and diversity.

The stronger separation of habitat types than urbanization levels in functional trait space, together with the greater descriptive variation in highly functionally rare species across W, WS, R, and FR habitats, indicates that the habitat‐classification scale can influence the functional differentiation detected in urban river corridors. Pooling adjacent habitats may obscure habitat‐associated patterns in functional rarity. The transferable implication is that habitat‐classification should retain ecologically meaningful transitional units and calibrate their boundaries to local hydrology, topography, and shoreline structure. This process and scale‐dependent approach is consistent with evidence that riparian management zones should be delineated according to local hydrology, topography, and vegetation functions rather than fixed‐width or universally applicable categories (Stutter et al. 2021). It also follows the principle that restoration objectives and success criteria should be developed with reference to site‐specific ecological conditions and measurable changes in ecosystem condition (Palmer et al. 2005). Future research should compare alternative habitat‐classification schemes across river systems using repeated surveys across multiple seasons and replicated restoration sites. These studies should also record site conditions, hydrological variation, restoration methods, and implementation history, because restoration responses may depend on physical constraints, implementation procedures, and the time elapsed since intervention (Burgess et al. 2023).

The context‐dependent patterns of functional rarity indicate that it should be interpreted in relation to urbanization level, lateral habitat context, and local site conditions. Management evaluations should compare ecologically comparable reference and altered habitat units matched for hydrological, physical, and urban‐context conditions and set context‐specific targets for species richness, functional rarity, and bank configuration (McManamay et al. 2018). Because this study did not evaluate interventions or temporal change, these management implications require testing through adaptive management.

## 5 Conclusion

This study shows that urbanization and lateral hydrological gradients were associated with spontaneous plant communities in contrasting ways within urban river corridors. Urbanization was associated with lower species and functional diversity but higher functional rarity and community‐weighted mean plant height, whereas increasing water distance showed the opposite pattern. Functional composition was more distinct among lateral habitat types than among urbanization levels, and the relationship between species diversity and functional diversity varied across urbanization levels and habitat types. Together, these findings indicate that changes in vegetation in urban river corridors require taxonomic, functional, structural, and contextual indicators. Assessments that integrate the surrounding urban matrix with within‐corridor lateral habitat variation provide a more complete characterization of community responses.

The single‐season survey and observational design define the scope of inference: the SEM estimates statistical associations among the specified pathways and does not establish causal mechanisms. Hydrological, soil, vegetation‐management, and engineering variables that were not included in the models may also have influenced the observed patterns. Future research should combine multi‐season, long‐term monitoring across multiple river systems with measurements of these environmental drivers and spatially explicit analyses that test nonlinear and scale‐dependent associations and alternative networks of community change. Probabilistic models can quantify uncertainty and variation across sites, habitats, and urban contexts, thereby strengthening tests of the generality of the observed patterns and of restoration strategies in urban river corridors.

## Author Contributions

**Yuxuan Li:** conceptualization (lead), data curation (lead), formal analysis (lead), investigation (lead), methodology (lead), visualization (lead), writing – original draft (lead), writing – review and editing (lead). **Yuandong Hu:** conceptualization (supporting), funding acquisition (lead), project administration (lead), resources (lead), supervision (lead), writing – original draft (supporting). **Yumeng Zhang:** data curation (supporting), formal analysis (supporting), writing – review and editing (supporting).

## Funding

The authors have nothing to report.

## Conflicts of Interest

The authors declare no conflicts of interest.

## Data Availability

The data supporting the findings of this study are publicly available in the Dryad Digital Repository (https://doi.org/10.5061/dryad.0vt4b8hf2). All relevant data and code necessary to reproduce the analyses are included in the repository and the supplementary files.
